# Independent and Joint Effects of Prenatal Incense-Burning Smoke Exposure and Children’s Early Outdoor Activity on Preschoolers’ Obesity

**DOI:** 10.3390/toxics12050329

**Published:** 2024-04-30

**Authors:** Meimei Chen, Esben Strodl, Weikang Yang, Xiaona Yin, Guomin Wen, Dengli Sun, Danxia Xian, Yafen Zhao, Weiqing Chen

**Affiliations:** 1 Department of Epidemiology, School of Public Health, Sun Yat-sen University, Guangzhou 510080, China; chenmm29@mail2.sysu.edu.cn; 2 School of Psychology and Counselling, Queensland University of Technology, Brisbane, QLD 4059, Australia; e.strodl@qut.edu.au; 3 Women’s and Children’s Hospital of Longhua District of Shenzhen, Shenzhen 518110, China; 4 School of Health Management, Xinhua College of Guangzhou, Guangzhou 510080, China

**Keywords:** incense-burning smoke, early outdoor activity, joint effect, obesity, preschool child

## Abstract

Incense burning is a significant source of indoor air pollution in many Asian regions. There is emerging evidence that maternal prenatal exposure to incense-burning smoke may be a risk factor for childhood obesity. We aimed to extend this new line of research by investigating the independent and joint effect of incense-burning smoke exposure, and children’s outdoor activity in early life, on preschoolers’ obesity. A total of 69,637 mother–child dyads were recruited from all kindergartens in the Longhua District of Shenzhen, China. Information on sociodemographic characteristics, maternal exposure to incense-burning smoke (IBS) during pregnancy, and frequency and duration of outdoor activity at the age of 1–3 years was collected by a self-administered questionnaire. In addition, the heights and weights of the children were measured by the research team. Logistic regression models and cross-over analyses were conducted to investigate the independent and combined effects of maternal exposure to incense-burning smoke during pregnancy and children’s early outdoor activity on obesity in preschoolers. We found that prenatal exposure to incense-burning smoke increased the risk of the presence of obesity in preschoolers’ (AOR = 1.13, 95% CI = 1.03–1.23). Additionally, lower frequencies (<3 times/week) or shorter durations (<60 min/time) of outdoor activity from the age of 1–3 years were significantly associated with the presence of obesity, with AORs of 1.24 (95% CI =1.18–1.32) and 1.11 (95% CI = 1.05–1.17), respectively. Furthermore, the cross-over analysis showed that prenatal exposure to IBS combined with a lower frequency of early outdoor activity (AOR = 1.47, 95% CI = 1.31–1.66) or a shorter duration of outdoor activity during ages of 1–3 years (AOR = 1.22, 95% CI = 1.07–1.39) increased the risk of obesity in preschoolers. Finally, additive interactions between prenatal exposure to IBS and postnatal outdoor activity on obesity were identified. Our study indicates that maternal exposure to incense-burning smoke during pregnancy and early lower postanal outdoor activity may independently and jointly increase the risk of obesity among preschoolers.

## 1. Introduction

The prevalence of childhood overweight and obesity has reached alarmingly high levels in the past decades worldwide. Globally, 124 million children and adolescents aged 5–19 years are affected by obesity, with the prevalence climbing from 0.7% to 5.6% in girls and from 0.9% to 7.8% in boys from 1975 to 2016, respectively [1]. In addition, according to the World Health Organization, approximately 38.2 million children under the age of five years were overweight or obese in 2019 [2]. According to the Global Burden of Disease study in 2015, China has the highest number of obese children in the world [3]. These statistics raise significant public health concerns given that there is mounting evidence that childhood obesity is a risk factor for numerous physical and mental health conditions in children [4,5] and that the persistence of obesity into adulthood is a significant risk factor for chronic diseases, such as diabetes, hypertension, hyperlipidemia [6], cardio cerebral vascular disease [7], and certain tumors in adults [8]. As such, early childhood is a critical period for preventing persistent obesity [9,10] and the subsequent health conditions associated with it. To guide the development of targeted public health interventions, it is therefore essential to identify significant risk and protective factors for childhood obesity.

While the etiology of childhood obesity is not yet fully understood, it is well known that childhood obesity is the result of a complex interplay of genetic, prenatal, and postnatal environmental factors. Moreover, there is increasing evidence showing that the programming of obesity begins prenatally and early postnatally [11]. For instance, previous research has identified the following prenatal risk factors for offspring obesity: high maternal prepregnancy BMI, maternal excessive weight gain during pregnancy, gestational diabetes, maternal physical activity during pregnancy, maternal smoking in pregnancy [12,13,14,15,16], and prenatal exposure to air pollution [17,18], as well as adverse birth outcomes of preterm birth, low birth weight, and macrosomia [19,20]. Additionally, prior studies have shown that early infants’ and toddlers’ health behaviors, such as nonbreastfeeding, consumption of high-sugar foods, fast eating pace, excessive screen time, and lack of physical activity, are risk factors for childhood obesity [19,20,21,22,23]. Conversely, playing outdoors, particularly in natural play spaces, boosts children’s physical activity, decreasing childhood obesity [24].

Based on the theory of developmental origins of health and disease (DOHaD), fetuses exposed to malnutrition and environmental hazards can experience heightened susceptibility to fetal origin of adult disease (FOAD), with this susceptibility further enhanced by postnatal exposure to hazardous environmental factors [25,26]. It is now widely accepted that exposure to hazardous in utero environments plays significant roles in fetal development and initiation of FOAD that may involve epigenetic alterations in the development of FOAD [25,26]. For example, Rueda-Clausen et al. found that prenatal hypoxia and postnatal high-fat diets in offspring were associated with an increased myocardial susceptibility to ischemia [27]. In addition, our recent study found that prenatal mosquito coil smoke exposure and early postnatal nutrition status can independently and jointly increase the risk of preschoolers’ obesity [28].

Incense burning, a practice widely observed in Asia and the Middle East, has been identified as a potential source of indoor air pollution, with the burning of incense releasing enormous levels of fine particles, polycyclic aromatic hydrocarbon (PAH), high concentrations of detrimental gases, and other toxic chemical compounds [29,30,31,32]. Previous studies have reported the potentially harmful health effects associated with incense burning, such as respiratory problems [33], neurobehavioral development [34], cardiovascular mortality [35], cancer [36,37], and adverse birth outcomes [38]. However, few studies have investigated the effect of exposure to incense burning on obesity. A cross-sectional study in Guangzhou reported that children exposed to three or more types of indoor air pollutants (cooking oil fumes, home decoration, secondhand smoke, and incense burning) had higher obesity anthropometric indices and increased odds of being overweight/obese [39]. However, the study found no statistical significance between incense burning and overweight/obesity. As such, there is a need for further research to clarify the key prenatal and postnatal factors linking exposure to indoor air pollutants with different childhood obesity measures.

Given the emerging evidence that exposure to indoor air pollution may be a risk factor for early childhood obesity, this study aimed to test the theory of DOHaD by examining whether maternal prenatal exposure to incense-burning smoke and early childhood physical activity levels independently and jointly predict the presence of childhood obesity in preschoolers. The aims of this study are important not only in terms of identifying significant risk factors for childhood obesity but also in terms of providing evidence to support the hypothesis that childhood health conditions are affected by an interaction of both prenatal and postnatal factors. Identifying these complex interactions is critical to enhancing the sophistication of our understanding of the biopsychosocial factors contributing to childhood ill health, thereby improving the effectiveness of public health interventions.

## 2. Materials and Methods

### 2.1. Study Population

**Figure 1:**
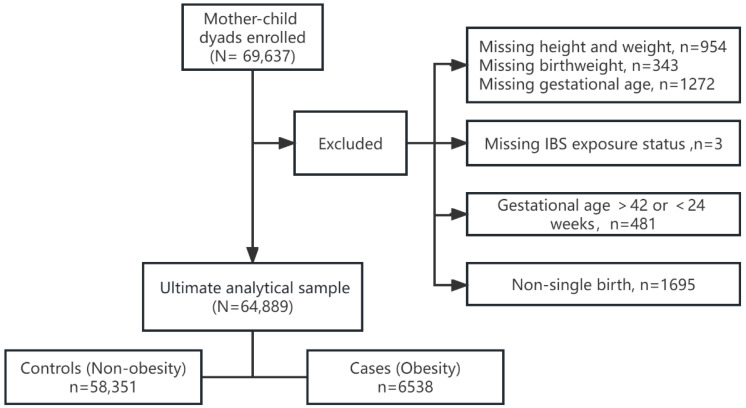
Flow diagram of the participant selection.

The Longhua Child Cohort Study (LCCS) is an ongoing prospective study that has involved the administration of a population-based survey once a year in the Longhua District of Shenzhen since September 2014. The aim of this study is to examine the influence of environmental factors during a preschooler’s early life on childhood psychobehavioral development. The LCCS recruits children when they enter preschool and invites their mothers to complete a self-administered structured questionnaire every year. A total of 69,637 child–mother dyads were recruited during the LCCS 2021 survey. After excluding children (1) with missing information on important sociodemographic characteristics (i.e., birth weight, gestational age, and present height and weight); (2) whose mothers did not report information of exposure to IBS during pregnancy; and (3) who were nonsingleton births and whose gestational age was low (under 24 weeks) or high (42 weeks or greater) (data from 64,889 (93.2%) child–mother dyads were included in this study (Figure 1)). We employed multiple imputation (MI) to fill in the missing data or outliers for covariates among 8868 (13.7%) participants whose questionnaires did not contain information on at least one chosen covariate.

This study was approved by the Ethics Committee of the School of Public Health at Sun Yat-Sen University and was conducted in accordance with the Declaration of Helsinki. Written informed consent was obtained from mothers.

### 2.2. Data Collection

The mothers were asked to complete a self-administered structured questionnaire regarding the sociodemographic characteristics of the parents (family monthly income, age at childbirth, marital status, parental education level, mother’s height and weight before prepregnancy, weight gain during pregnancy), as well as the child’s age, sex, gestational age, birth weight, and present height and weight.

### 2.3. Prenatal IBS Exposure Measurement

In addition, the questionnaire included three questions for the mother regarding incense-burning smoke exposure during pregnancy: “Did your household have the habit of burning incense at home during your pregnancy in 1–13 weeks (the first trimester)”? (2) “Did your household have the habit of burning incense at home during your pregnancy in 14–27 weeks (the second trimester)”? (3) “Did your household have the habit of burning incense at home during your pregnancy after 28 weeks (the third trimester)”? [(the response format was “no”, “sometimes (1 time per week)”, “often” (at least 2 times per week)”,)]. If the answer was “sometimes” or “often”, then prenatal exposure to IBS was considered present. Moreover, to identify the potentially sensitive period, we divided the participants into 8 subgroups according to the different combinations of IBS exposure status (No or Yes) in each trimester.

### 2.4. Early Frequency and Duration of Outdoor Activity Measurement

The frequency and duration of outdoor activity of children were evaluated by the following questions: (1) How often did your baby go outdoors for physical activity during the year of 1–3 years old? [(the response options were “0 = ≥3 times/week”, or “1 = <3 times/week”,)]; (2) How much time did your baby spend outdoors for physical activity on average during the year of 1–3 years old? [(the possible responses were 0 = ≥60 min/time”, or “1 = <60 min/time”,)]. The answers were transformed into the following variables that illustrated the children’s outdoor activity from birth to three years old: (1) the frequency of outdoor activity and (2) the duration of outdoor activity.

### 2.5. Measurement and Definition of Obesity

Children’s height and weight were measured by skilled nurses at the Longhua Maternity and Child Healthcare Hospital. A portable electronic weight scale (fractional value = 0.01 kg), placed on the ground level, was used to measure the weight of each preschooler, who was required to stand in the center of the scale bareheaded, barefooted, and dressed in close-fitting light clothes. The value was read and accurately recorded to 0.1 kg by nurses after stabilizing. A column human altimeter (fractional value = 0.1 cm), that was placed vertically against the wall on horizontal ground, was used to measure the weight of the preschoolers with each asked to stand on the pedal, with their heels close together, feet spaced at an angle of 60 degrees, chest raised, abdomen pulled in, and eyes looking straight ahead. With their line of sighting the same height as the slide board, the measurements were read by slider to the apex of the measured child’s skull.

The body mass index (BMI) was determined by dividing the weight in kilograms by the square of height in meters (kg/m^2^). Childhood obesity was defined as having a BMI higher than the specified thresholds based on age and gender, as per the BMI growth curves designed for Chinese children [40].

### 2.6. Potential Confounding Variables

In light of previously published studies [16,39,41], the potential covariates selected were children’s gender and age, birth weight, single child or not, premature birth, feeding pattern, early nutritional status, parental age at conception, maternal marital status and prepregnancy BMI, family income, maternal folic acid intake during pregnancy, prenatal exposure to environmental tobacco smoke, mosquito coil smoke, and cooking oil fumes.

### 2.7. Statistical Analyses 

Frequencies and proportions were used to describe the categorical variables, and the chi-square test was applied to test and describe associations of subjects’ characteristics with childhood obesity.

A series of binary logistic regression analyses were utilized to evaluate the independent and joint associations of prenatal exposure to incense-burning smoke, postnatal early frequency, and duration of outdoor activity with obesity in preschoolers after adjusting for the aforementioned covariates. The multiplicative interaction was examined by the interaction of odds ratio (IOR) in the logistic regression models. If the 95% CI of the IOR spanned 1, the multiplicative interaction was considered nonsignificant. Furthermore, relative excess risk due to interaction (RERI) and attributable proportion due to interaction (AP) were calculated. If the 95% CIs of RERI and AP did not span 0, the additive interaction was considered significant [42]. A cross-over analysis was performed to elucidate the possible sensitivity period. Additionally, sensitivity analyses were conducted after excluding children who were preterm birth (gestational age < 37 weeks) or low birth weight (birth weight < 2500 g). 

All statistical analyses were performed using R Studio version 4.3.1, with two-sided *p* < 0.05 deemed significant.

## 3. Results

### 3.1. Population Characteristics

Table 1 describes the comparison of demographic characteristics between obese and nonobese children. Of the 64,889 preschoolers included in this study, 6538 (10.0%) of them were obese, with the prevalence of obesity in boys being 11.7% and 8.2% in girls. Of the total sample, 6117 (9.4%) mothers reported that they had experienced prenatal exposure to IBS. Obese and nonobese preschoolers differed significantly on sex, age, single child or not, family income, marital status, maternal prepregnancy BMI, maternal gained weight during pregnancy, maternal folic acid intake during pregnancy, prenatal exposure to mosquito coil smoke and cooking oil fumes, education level of parents, feeding pattern, early nutritional status, birth weight, gestational age, and preterm birth or not.

### 3.2. Association between Maternal Exposure to IBS during Pregnancy and Obesity among Preschoolers

As shown in Table 2, compared with the nonexposed group, maternal exposure to IBS during the whole pregnancy significantly increased the risk of offspring obesity after controlling for a range of confounding factors (AOR = 1.13, 95% CI = 1.03~1.23). However, when the pregnancy was divided into three trimesters, there was a nonsignificant trend for maternal exposure to IBS in specific trimesters (the 1st trimester: AOR = 1.14, 95% CI = 0.96~1.34; the 2nd trimester: AOR = 1.19, 95% CI = 0.94~1.51; the 3rd trimester: AOR = 0.84, 95% CI = 0.68~1.04)

With regard to the frequency of IBS exposure during the different trimesters, after controlling for confounding factors, only maternal prenatal exposure to IBS ≥ 2 times/week in the 1st trimester (AOR = 1.31, 95% CI = 1.01~1.70) was significantly associated with an increased the risk of childhood obesity. Nonsignificant trends were found for the associations between those prenatally exposed to IBS with 1 time/week in the 1st trimester (AOR = 1.11, 95% CI = 0.94~1.31) and with both 1 time/week (AOR = 1.19, 95% CI = 0.94~1.51) and 2 times/week (AOR = 1.20, 95% CI = 0.84~1.69) in the 2nd trimester increased the risk of childhood obesity (Table 3). 

After controlling for confounding factors, the crossover analysis revealed that prenatal exposure to IBS concurrently in the 1st and 2nd trimesters (AOR = 1.50, 95% CI = 1.07~2.07) and in the whole three trimesters (AOR = 1.12, 95% CI = 1.01~1.25) significantly increased the risk of offspring obesity (Table 4). 

### 3.3. Association of Frequency and Duration of Outdoor Activity from 1 to 3 Years of Age with Children Obesity 

Table 5 presents the associations of the frequency and duration of outdoor activity from 1 to 3 years of age with children obesity. After adjusting for confounding factors, compared with children whose mothers reported that their child frequently went outdoors (≥3 times/week) at the age of 1~3 years, those who had a paucity of frequent outdoor activity had significantly greater odds of experiencing obesity during preschool (AOR = 1.24, 95% CI = 1.18~1.32). Additionally, a shorter duration of outdoor activity (<60 min/time) (AOR = 1.11, 95% CI = 1.05~1.17) was significantly associated with a higher risk of offspring obesity compared with children who had a longer duration of outdoor activity. Furthermore, compared with children with both the higher frequency (≥3 times/week) and the longer duration of outdoor activity at the age of 1~3 years, those with lower frequencies and longer durations, as well as those with lower frequencies (AOR = 1.13, 95% CI = 1.04~1.21) or with shorter durations of outdoor activity (AOR = 1.36, 95% CI =1.05~1.17), were significantly associated with preschool obesity.

### 3.4. Combination Effect between Maternal IBS Exposure during Pregnancy and Outdoor Activity from 1 to 3 Years of Age on Preschool Obesity

Table 6 presents the combined effect of maternal IBS exposure during pregnancy and outdoor activity during 1 to 3 years old on children’s obesity. 

Compared with children with no prenatal maternal IBS exposure and a higher frequency of outdoor activity, children with a combination of no prenatal exposure to IBS and lower frequencies of outdoor activity (AOR = 1.22, 95% CI = 1.07~1.39) and a combination between prenatal exposure to IBS and lower frequencies of outdoor activity (<60 min/time) (AOR = 1.47, 95% CI = 1.31~1.66) were both significantly more likely to experience obesity during preschool. Furthermore, there was a significant multiplicative interaction between prenatal maternal exposure to IBS and less frequent outdoor activity (<3 times/week) on obesity, with an IOR of 1.21 (95% CI = 1.01, 1.42). We also found a significant additive interaction between the frequency of outdoor activity and IBS exposure, with an RERI of 0.08 (95% CI = 0.03~0.13) and AP of 0.06 (95% CI = 0.03~0.09).

Compared with children with no prenatal IBS exposure and a longer duration of outdoor activity (≥60 min/time), children’s obesity was significantly associated with a combination of no prenatal exposure to IBS and shorter durations of outdoor activity (<60 min/time) (AOR = 1.11, 95% CI = 1.05~1.17), a combination of prenatal exposure to IBS and longer durations of outdoor activity (≥60 min/time) (AOR = 1.14, 95% CI = 1.02~1.27), and a combination of prenatal exposure to IBS and shorter durations of outdoor activity (<60 min/time) (AOR = 1.22, 95% CI = 1.07~1.39). Similarly, we found a significant additive interaction between prenatal maternal exposure to IBS and shorter durations of outdoor activity on obesity with an RERI of 0.07 (95% CI = 0.03~0.11) and an AP of 0.05 (95% CI = 0.03~0.08). 

### 3.5. Sensitivity Analysis

Sensitivity analyses were performed after excluding participants who were preterm birth or low birth weight (n = 13,079), and the main results are still similar to the aforementioned findings. More details are shown in Tables S1–S6.

## 4. Discussion

To the best of our knowledge, this is the first large-scale study on the association between maternal exposure to incense-burning smoke during pregnancy and obesity in Chinese preschoolers. Our results identify a significant association between maternal exposure to IBS during pregnancy and offspring obesity with a dose-response relationship between frequency of trimester-specific IBS exposure and children’s obesity. That is, mothers exposed to prenatal IBS in both the first and second trimesters concurrently and in all three trimesters were more likely to have offspring who experienced obesity in preschool. In addition, we observed that a low level of early outdoor activity was associated with a higher risk of obesity in preschoolers. Importantly, we also found both a multiplicative and an additive interaction between maternal prenatal IBS exposure and lower frequency of outdoor activity of children from 1 to 3 years old. Similarly, we found an additive interaction between maternal prenatal IBS exposure and a shorter duration of outdoor activity from 1 to 3 years old on the risk of childhood obesity.

In the past few decades, a series of studies have found associations between prenatal exposure to outdoor air pollution and childhood obesity. For instance, a prospective study in Boston found a positive relationship between exposure to ambient PM_2.5_ in utero, and from conception to 2nd years, and the risk of children between two and nine years being overweight and obese [43]. The Colorado-based Healthy Start study showed that second-trimester PM_2.5_ exposure was associated with a higher percent fat mass, and residential proximity to a highway during pregnancy was associated with higher odds of offspring being overweight at age 4~6 years [44]. Additionally, a study of 239 children born at ≥37 weeks gestation, from the Asthma Coalition on Community, Environment and Social Stress (ACCESS) project, indicated that increased PM_2.5_ exposure in early-to-mid pregnancy was more strongly associated with increased fat mass and higher BMI z-score in boys and with increased waist-to-hip ratio (WHR) in girls [45]. Recently, a review by Shi et al. concluded that exposure to air pollution during pregnancy might increase the risk of childhood obesity [17], while another review by Sun et al. indicated that perinatal exposure to PM_2.5_ could cause obesity in progeny [18]. Similarly, a systematic review by Qureshi et al. reported an association between prenatal exposure to environmental tobacco smoke and childhood obesity (OR = 1.91, 95% CI = 1.23~2.94) [46]. In line with these previous findings, our study found that maternal prenatal exposure to indoor air pollution of IBS was significantly linked with the presence of obesity in preschoolers, with a dose–response relationship between the frequency of trimester-specific IBS exposure in the 1st trimester and children obesity. Similarly, another recent study of ours found that prenatal exposure to indoor air pollution of mosquito coil smoke (MCS) was significantly positively associated with preschoolers’ obesity [28]. In this current study, the cross-over analysis highlighted the positive association of prenatal IBS exposure in the first and second trimesters concurrently and in the whole pregnancy with obesity in preschoolers, and it indicated a dose–response relationship between maternal exposure to IBS during pregnancy and offspring obesity. Based on the definition of the sensitivity period/critical window [47], given that no significant association was found for the first trimester after adjusting for covariates, we speculated that there may not exist a critical period for prenatal IBS exposure causing offspring obesity in childhood.

Obesity is typically considered as an abnormal or excessive fat accumulation caused by excessive energy intake or insufficient energy consumption. In the case of the same nutritional status, children with insufficient energy consumption are more likely to be obese. While limited studies reported the relationships between outdoor activity and childhood obesity, research has indicated that time spent outdoors is associated with increased physical activity, which has been reported to have a negative association with childhood obesity [48,49,50]. One study among children aged 10 to 12 found that for every additional hour spent outdoors, physical activity increased by 27 min a week, and the prevalence of overweight dropped from 41% to 27% [51]. The parents of preschool children reported that physical activity usually occurs during outdoor playtime as opposed to during indoor activities [52]. One systematic review revealed overall positive effects of outdoor time on physical activity compared with sedentary behavior [53]. Additionally, several researchers have proposed that increasing outdoor time could be an effective measure for limiting sedentary behavior and increasing physical activity and fitness in children [54,55]. The ecological model, as described by Davison et al., suggests that physical activity is one of the risk factors for children’s obesity [56]. The World Health Organization (WHO) recommends moderate-to-vigorous physical activity for children and adolescents for at least 60 min per day [57]. Consistent with these studies, we found that preschool children with less frequency or shorter durations of outdoor physical activity during 1–3 years old had a higher risk of obesity.

Consistent with the theory of DOHaD, the combination of maternal lifestyle during pregnancy and the early nutritional environment of the offspring is considered important for the development of obesity [16]. For example, an epidemiological study indicated that elevated exposure to mercury in utero was associated with a higher risk of the offspring being overweight or obese in childhood, with this risk being reduced by adequate maternal folate supplementation [58]. Additionally, an animal study suggested that prenatal dexamethasone and postnatal high-fat diet treatment caused dysregulation of nutrient-sensing molecules and circadian clock genes in visceral adipose tissue in rats [59]. Similarly, another animal study found that prenatal low-protein diets followed by postnatal high-fat diets resulted in a rapid increase in subcutaneous adipose tissue mass in the offspring, contributing to the development of obesity and insulin resistance [60]. In line with these findings, our study observed that children with mothers exposed to IBS during pregnancy had a much higher risk of obesity, especially when they experienced lower frequency or shorter durations of outdoor physical activity at ages of 1 to 3 years, with significant multiple and additive interactions between prenatal and postnatal variables. Taken together, all these findings proved the First-hit/Second-hit Framework of DOHaD [25,26]. 

Based upon the findings of prior studies, three possible pathways are proposed as explanations for the combination effect of prenatal IBS exposure and postnatal early childhood outdoor physical activity causing childhood obesity [25,61,62,63]. First, air pollution exposure may result in changes in metabolic hormones, such as leptin and adiponectin. A prospective study reported that maternal exposure to PM_2.5_ and NO_2_ during pregnancy increased umbilical cord blood adiponectin levels [64]. Second, oxidative stress and inflammation may be potential mediators. A Dutch study reported that maternal higher PM_10_ and NO_2_ exposure during pregnancy were associated with higher levels of C-reactive protein in cord blood at delivery [65]. Likewise, the Healthy Start study found that exposure to PM_2.5_ during midpregnancy had positive associations with maternal IL-6 [66]. Moreover, an animal study found that maternal supplementation with antioxidants reduced oxidative stress and prevented the offspring of Western-diet-fed rats from developing obesity [67]. Third, epigenetic modifications, involving histone modification, miRNA expression, and DNA methylation, are considered to be the most important mediators of developmental programming [25]. The ENVIRONAGE birth cohort found that PM_2.5_ exposure during the second trimester was negatively associated with DNA methylation of the lep promote status in the placenta, and with placental 3-NTp, a marker of oxidative/nitrosative stress [68]. Cai et al. reported that early pregnancy PM_10_ exposure was associated with placental DNA methylation involved in glucocorticoid metabolism in fetal growth [69]. These three pathways could contribute to changes in placental function and fetal intrauterine reprogramming, leading to a reduction in individual metabolic levels and increased susceptibility to obesity [25,55]. Based on this, insufficient fat consumption, such as a lack of outdoor exercise in early life, further exacerbates the development of obesity.

Some limitations should be considered when interpreting these results. First, the assessment of prenatal IBS exposure relied on retrospective self-reporting by mothers. This introduces the potential for recall bias, particularly concerning the accurate timing of exposure during specific trimesters. Second, the actual components and concentration of smoke emitted from burning incense, as well as the average duration of exposure to IBS during each period, were not objectively measured. Third, the recall time frame for mothers ranged from 2 to 7 years, so the validity of a mother’s recall regarding the frequency and duration of their child’s outdoor activities may have been biased. Fourth, all participants in this study were recruited from the Longhua District of Shenzhen, which may restrict the generalizability of our findings, as there could be variations in the habit of household incense burning across different cultures. Fifth, though a range of covariates were considered in our analysis, there were still unmeasured confounders, such as household ventilation conditions and outdoor air pollution conditions near the residential area, which may potentially influence the findings. Sixth, the assessment of outdoor activity relying on outdoor time and frequency, and the actual physical activity is not so clear, which may impact the findings. Seventh, retrospective studies provide relatively weaker evidence for establishing a causal relationship between the combined impact of prenatal and postnatal factors on childhood obesity. Therefore, it is necessary to conduct a prospective cohort to replicate these findings.

## 5. Conclusions

In summary, our findings suggest that maternal exposure to incense-burning smoke during pregnancy and low levels of postnatal outdoor physical activity in children, from the age of 1–3 years old, may independently and jointly increase the risk of obesity among preschoolers. In addition, there are dose–response relations between prenatal exposure to incense-burning smoke and childhood obesity. These findings highlight the necessity of implementing public health interventions to avoid maternal exposure to incense-burning smoke during pregnancy and to provide young children with more outdoor physical activity so that childhood obesity may be decreased.

## Figures and Tables

**Table 1:** Characteristics of the study participants.

Characteristics	Total (*N* = 64,889)	Obesity (*n* = 6538)	Prevalence (%)	*p*
Gender				<0.001
Boys	34,647	4070	11.7	
Girls	30,242	2468	8.2	
Age (years)				<0.001
<3	185	18	9.7	
3–4	12,527	1076	8.6	
4–5	22,313	2279	10.2	
≥5	29,864	3165	10.6	
Single child				<0.001
No	21,162	1759	8.3	
Yes	43,727	4779	10.9	
Family income (USD/month)				<0.001
<3077	32,246	3396	10.5	
3077~6154	21,781	2089	9.6	
≥6154	10,862	1053	9.7	
Marital status				<0.001
Married	63,225	6308	10.0	
Others	1664	230	13.8	
Maternal prepregnancy BMI				<0.001
Underweight (<18.5)	13,356	1059	7.9	
Normal (18.5~23.9)	45,976	4675	10.2	
Overweight (>24)	5557	804	14.5	
Maternal occupation				0.006
Full-time housewife	10,545	948	9.0	
others	54,344	5411	9.9	
Maternal gestational weight gain				<0.001
<5kg	3141	431	13.7	
5–10 kg	21,857	2223	10.2	
11–15 kg	25,598	2296	9.0	
16–20 kg	10,921	1168	10.7	
>20 kg	3372	420	12.5	
Folic acid intake during pregnancy				<0.001
No	38,198	4097	10.7	
Yes	26,691	2441	9.1	
Environmental tobacco smoke (ETS)				0.121
No	53,922	5388	10.0	
Yes	10,967	1150	10.5	
Mosquito coil smoke (MCS)				<0.001
No	45,092	4352	9.7	
Yes	19,797	2186	11.0	
Home renovated				0.026
No	61,363	6194	10.1	
Yes	2131	185	8.7	
Unclear	1395	159	11.4	
Cooking oil fumes				<0.001
No	13,597	1498	11.0	
Yes	51,292	5040	9.8	
Heavy metal exposure				0.831
No	64,392	6486	10.1	
Yes	497	52	10.5	
Benzene exposure				0.155
No	64,438	6483	10.1	
Yes	451	55	12.2	
Maternal education level				<0.001
Junior high school or lower	9498	1063	11.2	
High school	13,117	1391	10.6	
College or higher	42,274	4084	9.7	
Paternal education level				<0.001
Junior high school or lower	8532	980	11.5	
High school	13,256	1402	10.6	
College or higher	43,101	4156	9.6	
Maternal age at childbirth (years)				0.559
<35	58,481	5879	10.1	
≥35	6408	659	10.3	
Paternal age at childbirth (years)				0.03
<35	39,847	3926	9.9	
≥35	25,042	2612	10.4	
Feeding pattern				0.007
Breastfeeding	38,094	3864	10.1	
Artificial feeding	6424	706	11.0	
Mixed feeding	20,371	1968	9.7	
Nutritional status at 0–1 years old				<0.001
Poorly nourished	856	77	9.0	
Medium-nourished	15,949	13,689	8.6	
Well-nourished	48,084	5093	10.6	
Nutritional status at 1–3 years old				<0.001
Poorly nourished	996	81	8.1	
Medium-nourished	20,520	1592	7.8	
Well-nourished	43,373	4865	11.2	
Birth weight (g)				<0.001
<2500	3022	335	11.1	
2500–4000	55,763	5351	9.6	
≥4000	6104	852	14.0	
Preterm birth				<0.001
No	60,309	5982	9.9	
Yes	4580	556	12.1	

**Table 2:** Associations of maternal IBS exposure during pregnancy with children’s obesity.

Prenatal IBS Exposure	Total (*N* = 64,889)	Obesity (*n*, %)	AOR (95% CI) ^a^	AOR (95% CI) ^b^
The whole pregnancy				
No	58,772	5837 (9.9)	1.00	1.00
Yes	6117	701 (11.5)	1.13 (1.03, 1.23) **	1.13 (1.03, 1.23) **
1st trimester				
No	59,732	5933 (9.9)	1.00	1.00
Yes	5157	605 (11.7)	1.15 (1.05, 1.26) **	1.14 (0.96, 1.34)
2nd trimester				
No	60,243	5999 (10.0)	1.00	1.00
Yes	4646	539 (11.6)	1.14 (1.03, 1.26) **	1.19 (0.94, 1.51)
3rd trimester				
No	60,222	6019 (10.0)	1.00	1.00
Yes	4667	519 (11.1)	1.09 (0.99, 1.20)	0.84 (0.68, 1.04)

Model a: adjusted for child’s gender and age, birth weight, single child or not, premature birth, parental age at conception, maternal marital status and prepregnancy BMI, family income, prenatal exposure to ETS, MCS, cooking oil fumes, child’s feeding pattern, early nutritional status, folic acid intake during pregnancy. Model b: 1st trimester: adjusted for Model a + prenatal exposure to 2nd trimester and 3rd trimester; 2nd trimester: adjusted for Model a + prenatal exposure to 1st trimester and 3rd trimester; 3rd trimester: adjusted for Model a + prenatal exposure to 1st trimester and 2nd trimester. ** *p* < 0.01.

**Table 3:** Associations of frequency of trimester-specific IBS exposure during pregnancy with children obesity.

Frequency of IBS Exposure	Total (*N* = 64,889)	Obesity (*n*, %)	AOR (95% CI) ^a^	AOR (95% CI) ^b^
1st trimester				
Never	59,732	5933 (9.9)	1.00	1.00
1 time/week	4481	516 (11.5)	1.12 (1.01, 1.24) *	1.11 (0.94, 1.31)
≥2 times/week	676	89 (13.2)	1.33 (1.06, 1.67) *	1.31 (1.01, 1.70) *
2nd trimester				
Never	60,243	5999 (10.0)	1.00	1.00
1 time/week	4094	474 (11.6)	1.14 (1.03, 1.26) *	1.19 (0.94, 1.51)
≥2 times/week	552	65 (11.8)	1.15 (0.87, 1.48)	1.20 (0.84, 1.69)
3rd trimester				
Never	60,222	6019 (10.0)	1.00	1.00
1 time/week	4086	458 (11.2)	1.10 (0.99, 1.22)	0.85 (0.68, 1.06)
≥2 times/week	581	61 (10.5)	1.01 (0.76, 1.31)	0.77(0.54, 1.06)

Model a: adjusted for child’s gender and age, birth weight, single child or not, premature birth, parental age at conception, maternal marital status and prepregnancy BMI, family income, prenatal exposure to ETS, MCS, cooking oil fumes, child’s feeding pattern, early nutritional status, folic acid intake during pregnancy. Model b: 1st trimester: adjusted for Model a + prenatal exposure to 2nd trimester and 3rd trimester; 2nd trimester: adjusted for Model a + prenatal exposure to 1st trimester and 3rd trimester; 3rd trimester: adjusted for Model a + prenatal exposure to 1st trimester and 2nd trimester. * *p* < 0.05.

**Table 4:** Associations of trimester-specific IBS exposure during pregnancy with children obesity.

Trimester-Specific IBS Exposure			Total (*N* = 64,889)	Obesity (*n*, %)	OR (95% CI)	AOR (95% CI) ^a^
1st Trimester	2nd Trimester	3rd Trimester				
No	No	No	58,772	5837 (9.9)	1.00	1.00
Yes	No	No	980	118 (12.0)	1.24 (1.02, 1.50) *	1.16 (0.94, 1.40)
No	Yes	No	185	21 (11.4)	1.16 (0.72, 1.79)	1.10 (0.68, 1.71)
No	No	Yes	407	35 (8.6)	0.85 (0.59, 1.19)	0.87 (0.60, 1.21)
Yes	Yes	No	285	43 (15.1)	1.61 (1.15, 2.21) **	1.50 (1.07, 2.07) *
Yes	No	Yes	84	9 (10.7)	1.09 (0.51, 2.06)	1.08 (0.50, 2.06)
No	Yes	Yes	368	40 (10.9)	1.11 (0.78, 1.52)	1.13 (0.80, 1.56)
Yes	Yes	Yes	3808	435 (11.4)	1.17 (1.05, 1.30) **	1.12 (1.01, 1.25) *

^a^: adjusted for child’s gender and age, birth weight, single child or not, premature birth, parental age at conception, maternal marital status and prepregnancy BMI, family income, prenatal exposure to ETS, MCS, cooking oil fumes, child’s feeding pattern, early nutritional status, folic acid intake during pregnancy. * *p* < 0.05, ** *p* < 0.01.

**Table 5:** Associations of frequency and duration of outdoor activity from 1 to 3 years of age with children’s obesity.

Outdoor Activity during 1–3 Years Old	Total (*N* = 64,889)	Obesity (*n*, %)	OR (95% CI)	AOR (95% CI) ^a^
Frequency				
≥3 times/week	45,060	4261 (9.5)	1.00	1.00
<3 times/week	19,829	2277 (11.5)	1.24 (1.18, 1.31) ***	1.24 (1.18, 1.32) ***
Duration				
≥60 min/time	42,596	4175 (9.8)	1.00	1.00
<60 min/time	22,293	2363 (10.6)	1.09(1.03, 1.15) **	1.11 (1.05, 1.17) ***
Combination of Frequency and duration				
≥3 times/week + ≥60 min/time	32,402	3099 (9.6)	1.00	1.00
≥3 times/week + <60 min/time	12,658	1162 (9.2)	0.96 (0.89, 1.03)	0.98 (0.92, 1.06)
<3 times/week + ≥60 min/time	10,194	1076 (10.6)	1.12 (1.04, 1.20) **	1.13 (1.04, 1.21) **
<3 times/week + <60 min/time	9635	1201 (12.5)	1.35 (1.25, 1.44) ***	1.36 (1.26, 1.46) ***

^a^: adjusted for child’s gender and age, birth weight, single child or not, premature birth, parental age at conception, maternal marital status and prepregnancy BMI, family income, prenatal exposure to ETS, MCS, cooking oil fumes, child’s feeding pattern, early nutritional status, folic acid intake during pregnancy. ** *p* < 0.01, *** *p* < 0.001.

**Table 6:** Combination effect between maternal IBS exposure during pregnancy and outdoor activity from 1 to 3 years of age on children’s obesity.

IBS Exposure	Outdoor Activity	AOR (95% CI) *	IOR (95% CI)	RERI (95% CI) *	AP (95% CI) ^a^
Pregnancy	Frequency				
No	≥3 times/week	1.00			
No	<3 times/week	1.21 (1.14, 1.29) ***			
Yes	≥3 times/week	1.01 (0.90, 1.14)			
Yes	<3 times/week	1.47 (1.31, 1.66) ***	1.21 (1.01, 1.42) *	0.08 (0.03, 0.13)	0.06 (0.03, 0.09)
Pregnancy	Duration				
No	≥60 min/time	1.00			
No	<60 min/time	1.11 (1.05, 1.17) ***			
Yes	≥60 min/time	1.14 (1.02, 1.27) *			
Yes	<60 min/time	1.22 (1.07, 1.39) **	0.96 (0.81, 1.14)	0.07 (0.03, 0.11)	0.05 (0.03, 0.08)

^a^: adjusted for child’s gender and age, birth weight, single child or not, premature birth, parental age at conception, maternal marital status and prepregnancy BMI, family income, prenatal exposure to ETS, MCS, cooking oil fumes, child’s feeding pattern, early nutritional status, folic acid intake during pregnancy. * *p* < 0.05, ** *p* < 0.01, *** *p* < 0.001.

## Data Availability

Due to participant privacy concerns, the datasets created and/or analyzed in this study cannot be accessed by the general public. However, they can be obtained from the corresponding author upon reasonable request.

## Supplementary Materials

The following supporting information can be downloaded at https://www.mdpi.com/article/10.3390/toxics12050329/s1, Table S1: Associations between maternal incense-burning smoke exposure during pregnancy and children’s obesity excluding PTB and LBW; Table S2: Associations between trimester-specific IBS exposure during pregnancy and children’s obesity excluding PTB and LBW; Table S3: Associations between frequency of trimester-specific IBS exposure during pregnancy and children’s obesity excluding PTB and LBW; Table S4: Associations between frequency of trimester-specific IBS exposure during pregnancy and children’s obesity excluding PTB and LBW; Table S5: Associations between frequency and duration of outdoor activity from 1 to 3 years of age on preschool obesity excluding PTB and LBW; Table S6: Combination Effect between maternal IBS exposure during pregnancy and outdoor activity from 1 to 3 years of age on preschool obesity.
