# Supplementary material for: Independent and Joint Effects of Prenatal Incense-Burning Smoke Exposure and Children’s Early Outdoor Activity on Preschoolers’ Obesity
Source: Toxics. 2024 Apr 30;12(5):329. doi: 10.3390/toxics12050329 (PMC11126066; doi:10.3390/toxics12050329)
Supplement: Supplementary file 1 [file toxics-12-00329-s001.zip › supplement_questionnaire.pdf]

Health Records of Preschool children in Longhua District,

Shenzhen (Excerpts)

Part I.Demographic Characteristics

O1 **Child name**: ___

O2 **Date of birth**: ___

O3 **Child sex**

1=Boy, 2=Girl

O4 **Id number: ___**

O9 **Home address: ___**

A2 **The child's height is ___ centimeters and his weight is___ kilograms.**

A5 **The child's mother's marital status is:**

1= Married, 2= Others

A6 **Mother's date of birth： ___**

A7 **Mother's degree is：**

1= junior high school and below, 2= high school, 3= junior college and above

A8 **Mother's occupation is:**

1= workers,2= farmers,3= soldiers,4= administrative cadres,5= science and technology, medical, teachers,6= individuals, business, enterprise, government (service personnel),7= housework,8= unemployed people,9= students,10= others

A10 **Father's date of birth： ___**

A11 **Father's degree is：**

1= junior high school and below, 2= high school, 3= junior college and above

A12 **Father's occupation is:**

1= workers,2= farmers,3= soldiers,4= administrative cadres,5= science and technology, medical, teachers,6= individuals, business, enterprise, government (service personnel),7= housework,8= unemployed people,9= students,10= others

A13 **The total monthly income of your family is**

1= < 20,000 yuan, 2= 20,000-40,000 yuan (including 20,000), 3= more than 40,000 yuan (including 40,000)

A15 **Is your child an only child?**

0= Yes, 1= No

Part II. Pregnancy Situation

B11.2 **Whether the mother took folic acid during pregnancy with this child**

0= No, 1= Yes

B12.2 **Whether the mother was exposed to interior decoration during the pregnancy of this child**

0= No, 1= Yes

B12.3 **Whether the mother exposed to heavy metals (lead, chromium, cadmium, etc.) during pregnancy with this child?**

0= No, 1= Yes

B12.4 **Whether the mother exposed to organic solvents (xylene, etc.) during pregnancy with this child?**

0= No, 1= Yes

B19 **The mother's height before this pregnancy was ___ centimeters and her weight was ___ kilograms**

B20 **The mother gained weight during pregnancy with this child:**

1=<5 kg,2=5-10 kg (inclusive of 10kg),3=10-15 kg (inclusive of 15kg),4=15-20 kg (inclusive of 20kg),5= > 20kg

During the pregnancy of this child, did anyone in the family living with the mother smoke in the presence of the
B21 **mother (including e-cigarettes)?**

0= No, 1= Yes

B27 **Was the mother exposed to cooking fumes during the first trimester of pregnancy?**

0= No contact,1= occasional contact,2= frequent contact,3= almost daily contact

B28 **Was the mother exposed to cooking fumes during the second trimester of pregnancy?**

0= No contact,1= occasional contact,2= frequent contact,3= almost daily contact

B29 **Was the mother exposed to cooking fumes during the third trimester of pregnancy?**

0= No contact,1= occasional contact,2= frequent contact,3= almost daily contact

Did your household have the habit of lighting mosquito-repellent incense (excluding electric mosquito-repellent
B31 **incense) during your pregnancy in 1-13 weeks (the first trimester)?**

0=no, 1= sometimes (1 times/week), 2=often (times/week).

Did your household have the habit of lighting mosquito-repellent incense (excluding electric mosquito-repellent
B32 **incense) during your pregnancy in 14-27 weeks (the second trimester)?**

0=no, 1= sometimes (1 times/week), 2=often (times/week).

Did your household have the habit of lighting mosquito-repellent incense (excluding electric mosquito-repellent
B33 **incense) during your pregnancy after 28 weeks (the third trimester)?**

0=no, 1= sometimes (1 times/week), 2=often (times/week).

Did your household have the habit of burning incense at home during your pregnancy in 1-13 weeks (the first
B34 **trimester)?**

0=no, 1= sometimes (1 times/week), 2=often (times/week).

Did your household have the habit of burning incense at home during your pregnancy in 14-27 weeks (the second
B35 **trimester)?**

0=no, 1= sometimes (1 times/week), 2=often (times/week).

Did your household have the habit of burning incense at home during your pregnancy after 28 weeks (the third
B36 **trimester)?**

0=no, 1= sometimes (1 times/week), 2=often (times/week).

Part III. Child birth

C1 **The gestational age of the child is ___ weeks**

C2 **Whether premature birth:**

0= no,1= yes

C3 **Weight at birth: ___ kg.**

BW Birth weight (cleaned for gestational age)

C4 **Length at birth: ___ cm.**

Part IV. 0-3 years old

D1 **Feeding method for children from 0 to 6 months**

1= Breastfeeding, 2= Breastfeeding, 3= Mixed feeding (refers to the above two feeding methods)

D5 **The nutritional status of children during the 0-1 years of age is as follows:**

1= Poor, 2= Medium, 3= Well

E1 **The nutritional status of children during the 1-3 years of age is as follows:**

1= Poor, 2= Medium, 3= Well

E10 **How often did your baby go outdoors during the year of 1-3 years old?**

0= < 3 times/week, 1=⩾3 times/week

E11 **How much time did your baby spend outdoors on average during the year of 1-3 years old?**

1=60 minutes/time, 2=⩾60 minutes/time
