# Supplementary material for: Independent and Joint Effects of Prenatal Incense-Burning Smoke Exposure and Children’s Early Outdoor Activity on Preschoolers’ Obesity
Source: Toxics. 2024 Apr 30;12(5):329. doi: 10.3390/toxics12050329 (PMC11126066; doi:10.3390/toxics12050329)
Supplement: Supplementary file 1 [file toxics-12-00329-s001.zip › supplement_sensitivity analysis.pdf]

Sensitivity analysis after excluding PTB and LBW

Table S1. Associations between maternal incense burning smoke exposure during pregnancy and children's obesity.

IBS exposure	AOR (95% CI) [a]
During pregnancy	
No	1.00
Yes	1.13 (1.03, 1.25) *

Model a: adjusted for child's gender and age, single child or not, parental age at conception, maternal marital status and pre-pregnancy BMI, family income, prenatal exposure to ETS, MCS, cooking oil fumes, child's feeding pattern, early nutritional status, folic acid intake during pregnancy.

AOR: adjusted OR. * $p < 0.05$, ** $p < 0.01$.

Table S2. Associations between trimester-specific IBS exposure during pregnancy and children's obesity.

Trimester-specific IBS exposure	Total, N = 64,889	Obesity, n (%)	Excluding PTB and LBW
			AOR (95% CI) [a]
**1st trimester**			
No	59732	5933 (9.9)	1.00
Yes	5157	605 (11.7)	1.15 (1.04, 1.28) **
**2nd trimester**			
No	60243	5999 (10.0)	1.00
Yes	4646	539 (11.6)	1.15 (1.04, 1.28) **
**3rd trimester**			
No	60222	6019 (10.0)	1.00
Yes	4667	519 (11.1)	1.08 (0.96, 1.20)

Model a: adjusted for child's gender and age, single child or not, parental age at conception, maternal marital status and pre-pregnancy BMI, family income, prenatal exposure to ETS, MCS, cooking oil fumes, child's feeding pattern, early nutritional status, folic acid intake during pregnancy.

Table S3. Associations between frequency of trimester-specific IBS exposure during pregnancy and children's obesity.

Frequency of IBS exposure	Excluding PTB and LBW
	AOR (95% CI) [a]
1st trimester	
Never	1.00
1 time/week	1.13 (1.01, 1.26) *
≥2 time/week	1.35 (1.04, 1.73) *
2nd trimester	

Never	1.00
1 time/week	1.14 (1.02, 1.28) *
≥2 time/week	1.25 (0.93, 1.64)
3rd trimester	
Never	1.00
1 time/week	1.08 (0.96, 1.21)
≥2 time/week	1.08 (0.79, 1.43)

Model a: adjusted for child's gender and age, single child or not, parental age at conception, maternal marital status and pre-pregnancy BMI, family income, prenatal exposure to ETS, MCS, cooking oil fumes, child's feeding pattern, early nutritional status, folic acid intake during pregnancy.

Table S4. The associations between frequency of trimester-specific IBS exposure during pregnancy and children's obesity.

group	Trimester-specific exposure			AOR (95% CI) [a]
	1st trimester	2nd trimester	3rd trimester	
1	No	No	No	1.00
2	Yes	No	No	1.18 (0.94, 1.45)
3	No	Yes	No	1.28 (0.77, 2.00)
4	No	No	Yes	0.85 (0.56, 1.23)
5	Yes	Yes	No	1.61 (1.12, 2.25) **
6	Yes	No	Yes	0.77 (0.27, 1.75)
7	No	Yes	Yes	0.69 (0.24, 1.56)
8	Yes	Yes	Yes	1.13 (1.01, 1.27) *

Model a: adjusted for child's gender and age, single child or not, parental age at conception, maternal marital status and pre-pregnancy BMI, family income, prenatal exposure to ETS, MCS, cooking oil fumes, child's feeding pattern, early nutritional status, folic acid intake during pregnancy.

AOR: adjusted OR. * $p < 0.05$, ** $p < 0.01$.

Table S5. Associations between frequency and duration of outdoor activity from 1 to 3 years of age on preschool obesity.

**Outdoor activity**	AOR (95% CI) [a]
Frequency during 1-3 years old (%)	
≥3times/week	1.00
<3 times/week	1.24 (1.17, 1.32) ***
Duration during 1-3 years old (%)	
≥60 min/time	1.00
<60 min/time	1.09 (1.03, 1.16) **
Overall outdoor factors	
≥ 3 times/week + ≥ 60 min/time	1.00
≥ 3 times/week + < 60 min/time	0.99 (0.91, 1.07)

< 3 times/week + ≥ 60 min/time	1.15 (1.06, 1.25) ***
< 3 times/week + < 60 min/time	1.33 (1.23, 1.45) ***

Model a: adjusted for child's gender and age, single child or not, parental age at conception, maternal marital status and pre-pregnancy BMI, family income, prenatal exposure to ETS, MCS, cooking oil fumes, child's feeding pattern, early nutritional status, folic acid intake during pregnancy.

AOR: adjusted OR. * $p < 0.05$, ** $p < 0.01$, *** $p < 0.001$.

Table S6. Combination Effect between maternal IBS exposure during pregnancy and outdoor activity from 1 to 3 years of age on preschool obesity.

IBS exposure	Outdoor activity	Excluding PTB and LBW			
		AOR (95% CI) [a]	IOR (95% CI)	RERI (95% CI) [a]	AP (95% CI) [a]
Prenatal exposure	Frequency during 1-3 years old				
No	≥3times/week	1.00			
No	<3 times/week	1.20 (1.13, 1.29) ***			
Yes	≥3times/week	1.01 (0.88, 1.15)			
Yes	<3 times/week	1.49 (1.31, 1.70) ***	1.23 (1.02, 1.49) *	0.09 (0.04,0.15)	0.07 (0.03,0.10)
Prenatal exposure	Duration during 1-3 years old				
No	≥60 min/time	1.00			
No	<60 min/time	1.09 (1.02, 1.16) **			
Yes	≥60 min/time	1.13 (1.00, 1.27)			
Yes	<60 min/time	1.23 (1.06, 1.42) **	1.00 (0.83, 1.21)	0.07 (0.03,0.12)	0.06 (0.03,0.09)

Model a: adjusted for child's gender and age, single child or not, parental age at conception, maternal marital status and pre-pregnancy BMI, family income, prenatal exposure to ETS, MCS, cooking oil fumes, child's feeding pattern, early nutritional status, folic acid intake during pregnancy.

AOR: adjusted OR. * $p < 0.05$, ** $p < 0.01$, *** $p < 0.001$.
